# Disc measurement and nucleus calibration in a smoothened lumbar model increases the accuracy and efficiency of *in*-*silico* study

**DOI:** 10.1186/s13018-021-02655-4

**Published:** 2021-08-13

**Authors:** Jingchi Li, Chen Xu, Xiaoyu Zhang, Zhipeng Xi, Shenglu Sun, Ke Zhang, Xiaoyang Fang, Lin Xie, Yang Liu, Yueming Song

**Affiliations:** 1 Department of Orthopedic Surgery and Orthopedic Research Institute, West China Hospital/West China School of Medicine for Sichuan University, Chengdu, 610041 China; 2 Department of Spine Surgery, Changzheng Hospital Affiliated to the Naval Medical University, Shanghai, 200041 China; 3 Department of Spine Surgery, Affiliated Hospital of Integrated Traditional Chinese and Western Medicine for Nanjing University of Chinese Medicine, Nanjing, 210028 China; 4 Department of Imaging, Affiliated Hospital of Integrated Traditional Chinese and Western Medicine for Nanjing University of Chinese Medicine, Nanjing, 210028 Jiangsu China

**Keywords:** Model calibration, Finite element analysis, Cross-sectional area ratio, Relative nucleus position, Smoothened surfaces

## Abstract

**Backgrounds:**

Finite element analysis (FEA) is an important tool during the spinal biomechanical study. Irregular surfaces in FEA models directly reconstructed based on imaging data may increase the computational burden and decrease the computational credibility. Definitions of the relative nucleus position and its cross-sectional area ratio do not conform to a uniform standard in FEA.

**Methods:**

To increase the accuracy and efficiency of FEA, nucleus position and cross-sectional area ratio were measured from imaging data. A FEA model with smoothened surfaces was constructed using measured values. Nucleus position was calibrated by estimating the differences in the range of motion (RoM) between the FEA model and that of an *in*-*vitro* study. Then, the differences were re-estimated by comparing the RoM, the intradiscal pressure, the facet contact force, and the disc compression to validate the measured and calibrated indicators. The computational time in different models was also recorded to evaluate the efficiency.

**Results:**

Computational results indicated that 99% of accuracy was attained when measured and calibrated indicators were set in the FEA model, with a model validation of greater than 90% attained under almost all of the loading conditions. Computational time decreased by around 70% in the fitted model with smoothened surfaces compared with that of the reconstructed model.

**Conclusions:**

The computational accuracy and efficiency of in-silico study can be improved in the lumbar FEA model constructed using smoothened surfaces with measured and calibrated relative nucleus position and its cross-sectional area ratio.

## Key points

- Irregular surfaces in models directly reconstructed based on imaging data may increase the computational burden and decrease the credibility of FEA.
- The nucleus position and its cross-sectional area ratio have been proven to affect biomechanical environment but its definition do not conform to a uniform standard.
- The computational accuracy and efficiency can be optimized by replacing irregular surfaces and determining a precise definition of above indicators.

## Introduction

With advancements in computational techniques, finite element analysis (FEA), a kind of digital mechanical simulation research method, has been promoted rapidly in spinal biomechanical studies for the study concerning deterioration of biomechanical environments, which are among the most important reasons for several kinds of spinal diseases and spinal surgical failures [1–9]. Current FEA modeling methods can reflect real situations following specific validation processes by evaluating the differences in the biomechanical indicators of FEA studies based on *in*-*vitro* biomechanical studies [5, 10–14]. However, model construction and calibration have limitations.

**Fig. 1:**
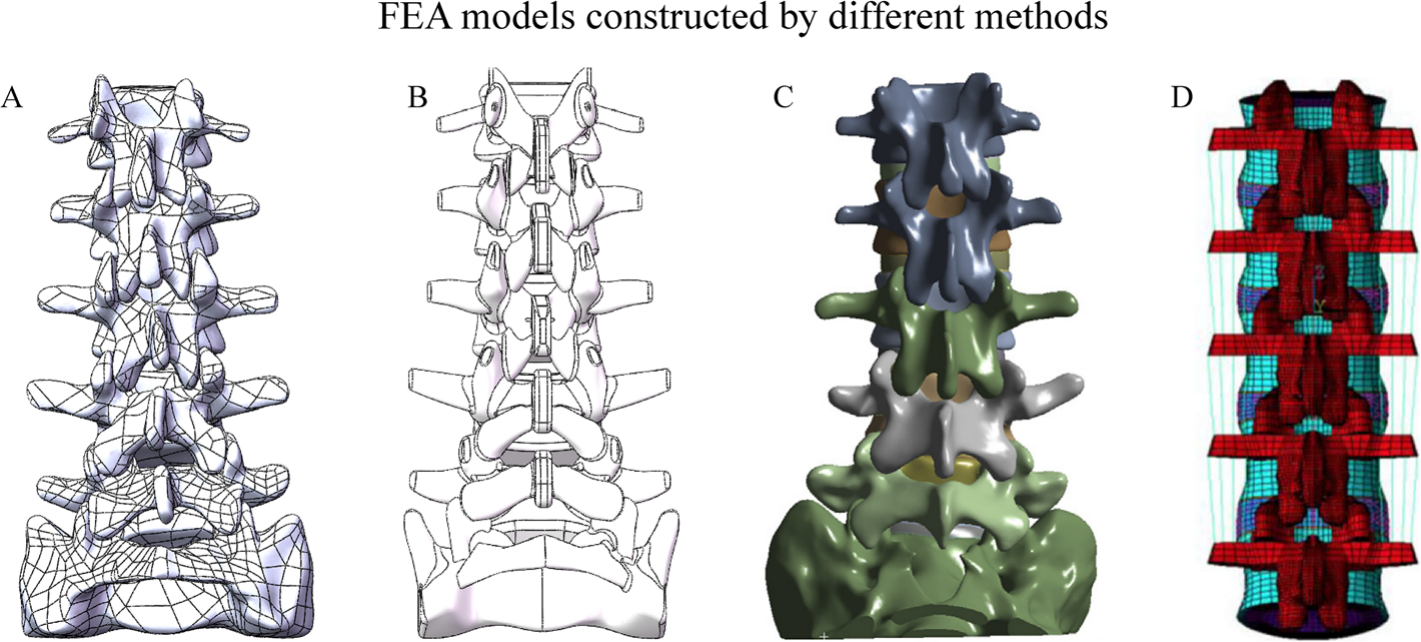
Schematic for different FEA model construction methods. **A** Model reconstructed directly. **B** Model constructed by fitted curves. **C** Model with irregular surfaces and structures in anterior published study [16]. **D** Over simplified model in anterior published study [18]

Models reconstructed on the basis of imaging data (this model reconstruction method is abbreviated to ‘model reconstructive method’ in the following passage) have numerous macroscopic irregular surfaces and structures [5, 11, 15, 16], which may increase the uncertainty of analysis results and reduce the repeatability of experiments [15, 16]. To solve this problem, standard geometries are selected for model construction in several studies (this model reconstruction method is abbreviated to ‘standard geometry method’ in the following passage) [4, 17, 18]. However, the resulting structural oversimplification is a visible limitation of FEA and may reduce the credibility of FEA for this model; as such, oversimplification cannot simulate the details of a model’s outline, especially in the posterior column (Fig. 1) [4, 18, 19].

**Fig. 2:**
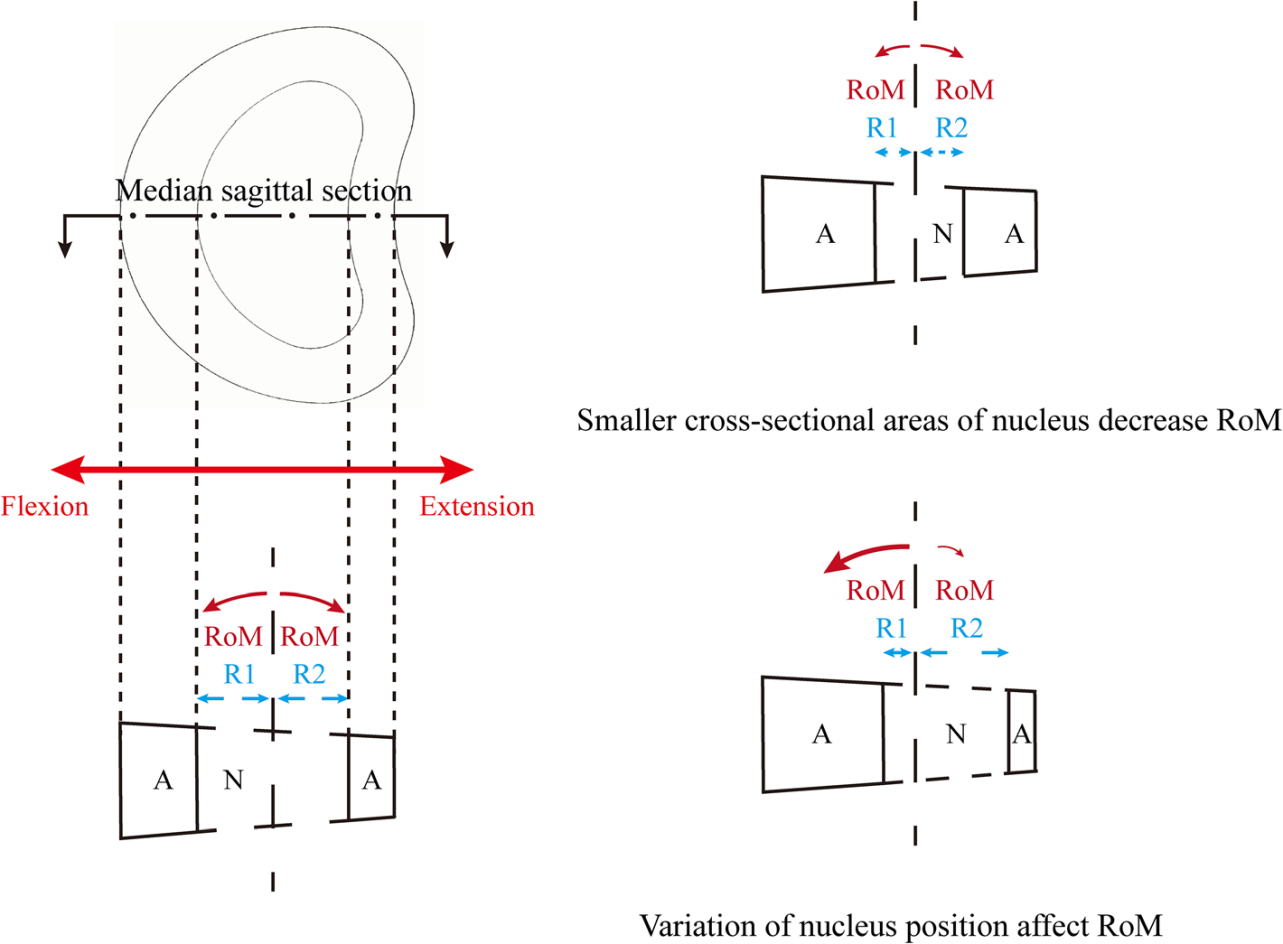
Effects of the variations in the nucleus cross-sectional areas and relative positions on the RoMs. The annulus can be approximated as two differently sized quadrangular annuls on a specific plane. M1 and M2 are set as the applied forward and backward torques (i.e., flexion and extension in the central section of the disc sagittal plane), R1 and R2 are the distances between the anterior and posterior parts perpendicular to the torque axis centre and edges of the nucleus, respectively. Changes in R1 and R2 originating from different nucleus positions and cross-sectional areas will induce changes in the RoM under the same M1 and M2. Furthermore, this change will extend to different sections if the force on the two-dimensional planar structure is extended to three-dimensional space A = Annulus; N = Nucleus

Besides, the relative nucleus position and its cross-sectional area should also be set clearly for which was shortly related to the biomechanical performance of FEA. The stiffness of the nucleus is obviously lower than that of the annulus [20, 21], so the stiffness of the intervertebral disc changes as the cross-sectional areas and relative position of the nucleus vary, and the related change in RoM can be deduced logically under the same sizes of moments [22, 23] (Fig. 2).

But the definition of such indicators has not been defined consistently in previous FEA studies. For example, Tang et al. modeled the center of the nucleus as being slightly posterior to the center of the disc, occupying 43% of the total disc volume [23]. Schmidt et al. modeled the center of the nucleus as being 3.5 mm toward the posterior side, with a nucleus size that was approximately 44% of the total disc area [24]. The lumbar model of Akiah et al. determined that the nucleus accounted for 33% of the disc volume [25], whereas this rate decreased to 30%, increased to 35%, 40%, and 50% in different reports separately [9, 26–29]. Moreover, this issue has not even been mentioned in the vast majority of published studies. Given that individual differences are inevitable among the discs in different models, the accurate definition of the relative nucleus position should be a ratio (such as a ratio of the anterior and posterior edges of the annulus to the relative nucleus edges) rather than an ambiguous description (e.g., ‘slightly posterior’) or a specific value (e.g., 3.5 mm).

In summary, the optimisation of FEA can serve as a basis for conducting further spinal biomechanical studies. This study was initiated to increase the accuracy and efficiency of FEA by replacing irregular surfaces from a reconstructed model with smoothened surfaces (on the basis of retaining its original outline) and defining the relative nucleus position and its cross-sectional area ratio accurately. An extensive literature search indicated that no similar studies have been published to date.

## Methods

### MRI measurements of the indicators

Three observers, two senior spine surgeons and a seasoned musculoskeletal radiologist, reviewed the lumbar MRI (Discovery MR750 3.0T, GE Healthcare; Chicago, Illinois, USA) data collected in our hospital over the past 3 years. Referring to the imaging protocol of published studies [30–32], the imaging protocol was presented as follows: a T2-weighted sequence (repetition time [TR]/echo time [TE]: 2500 ms/105 ms; field of view: 512 × 512; receiver bandwidth: variable; 4.5-mm slice with gap of 0.9 mm; number of excitations: 2). And as is reported previously, the border of the nucleus was identified as the boundary between high- and low-signal areas [33, 34].

The L4–L5 disc was selected owing to its high incidence rate of DDD [1–3]. T2-weighted imaging in the sagittal plane was selected to assess the grades of disc degeneration based on the work of Pfirrmann et al [30], with grade I and II degeneration considered indicative of a normal disc included in this study [21]. The MRI data were independently reviewed, and they were included in the analysis only when all observers confirmed that the disc was a normal disc. The kappa statistic was used to analyze the included imaging data to ensure homogeneity in the interobserver classification [33, 34].

**Fig. 3:**
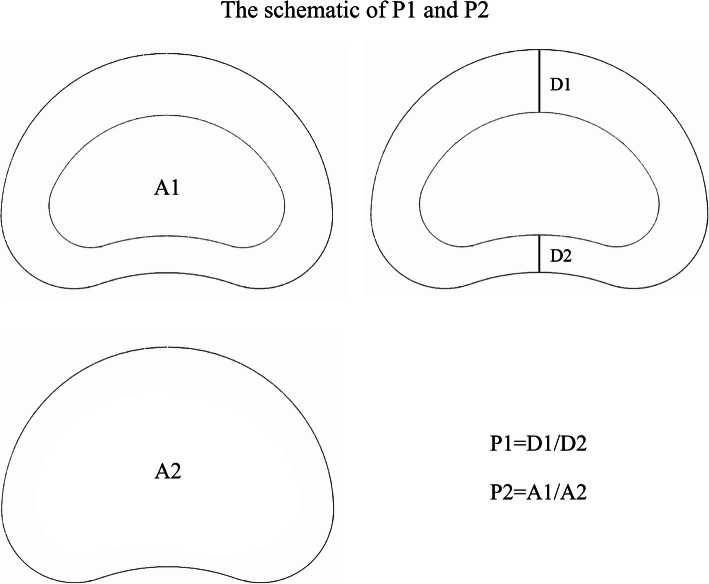
Schematic of P1 and P2

The distances from the anterior and posterior edges of the annulus to the nucleus edges are set as D1 and D2, respectively. The cross-sectional areas of the nucleus and disc are set as A1 and A2, respectively. The ratios between the mean values of D1 and D2 and A1 and A2 are set as P1 and P2, respectively (Fig. 3). Cronbach’s α reliability statistic was calculated to ensure the homogeneity of the measured values [35, 36].

### Calibration and validation of the FEA model

#### Construction of an intact model

Bone structures from L1 to S1, including cortical shells, cancellous cores, and posterior structures, were directly reconstructed on the basis of high-resolution computed tomography imaging data. The latter was obtained from a 24-year-old male volunteer with no history of lumbar diseases [6, 12, 35]. Reconstructed bone structures were used as templates of the subsequent model construction. The template was layered, and contours were set with the fitted curves on each layer to replace irregular surfaces and structures. The external contours of the new model were overlapped with the template. Compared with those of ‘model reconstructive’ and ‘standard geometry’ methods, this modeling method not only eliminates irregular surfaces and structures and strictly symmetric along the sagittal plane but also retains the outer contour of the structures from imaging data (i.e. reduce structural distortion) (Fig. 1).

**Fig. 4:**
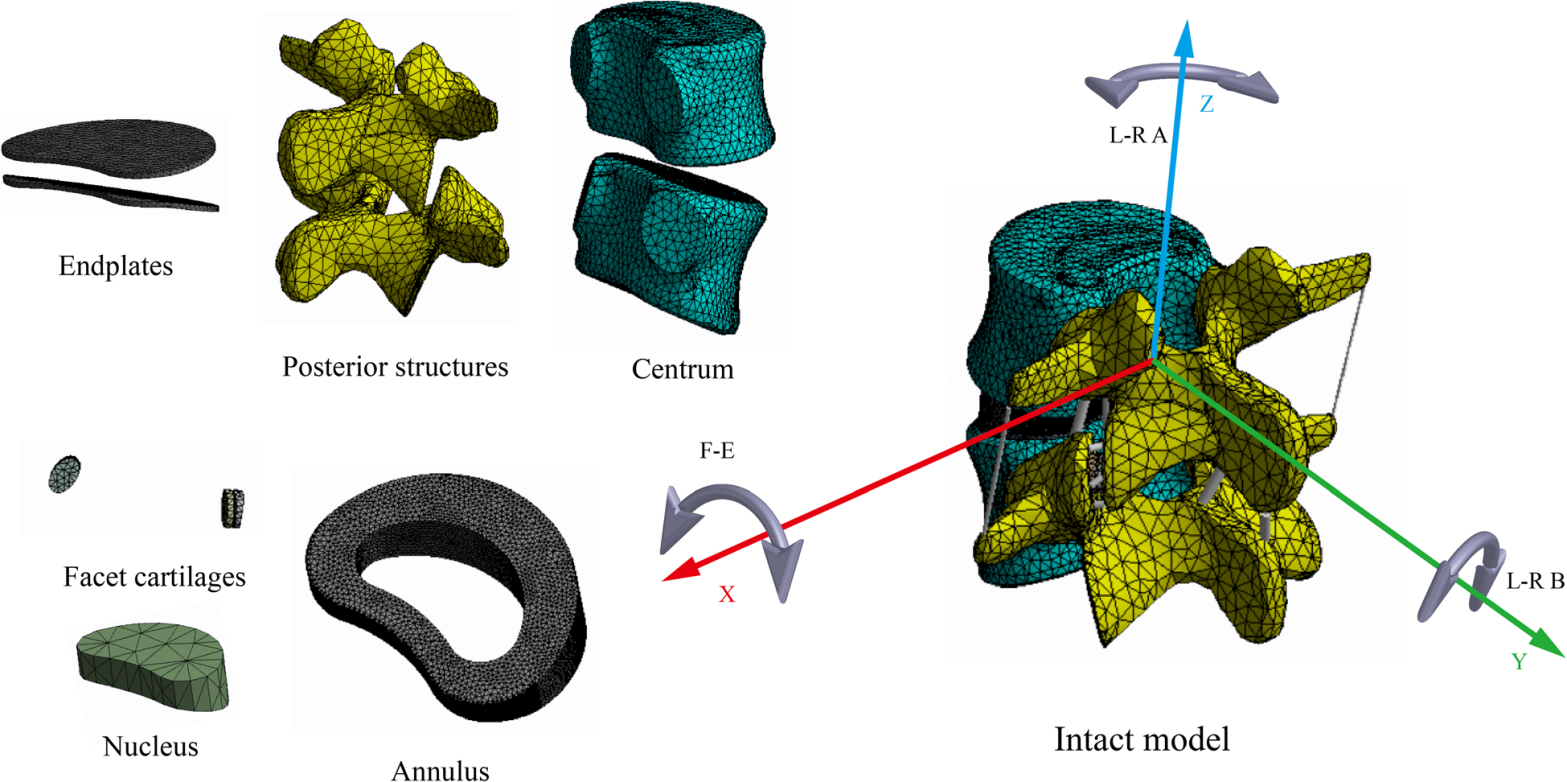
Intact model and components of the current models

To be consistent with the segment selection via MRI, the bone structures of the L4–L5 segments were selected, and the corresponding non-bone structures were constructed with the fitted curves; the facet joint gap was set as 0.5 mm. The centroid of the annulus outlines and the inferior surface of L4 were defined as the same point for the accurate placement of the annulus. Six different ligaments and a capsule of facet joints were constructed during the FEA preprocessing phase (Fig. 4) [5, 10, 37]. The definition of the relative nucleus position and its cross-sectional area ratio were confirmed according to P1 and P2, and the outer contour of the nucleus was obtained using the same ratio reduction as that of the disc contour to ensure that the intervertebral disc and nucleus have the same central point, for easy adjustment during the calibration process.

#### Boundary and loading conditions

Completely identical boundary conditions were used in the model calibration and validation processes. Tetrahedral and hexahedral elements with different sizes were selected during mesh generation (Fig. 4), with smaller sizes (mesh refinement) being used in areas that experienced serious mesh distortion, and the cartilage–cartilage contact was defined as frictionless [6, 12, 37]. Material properties of current models were defined as per our previously published studies [37, 38]. Six degrees of freedom were rigidly fixed under the inferior of L5, and moments were applied to the superior of L4 [21, 39, 40].

#### Model calibration

**Fig. 5:**
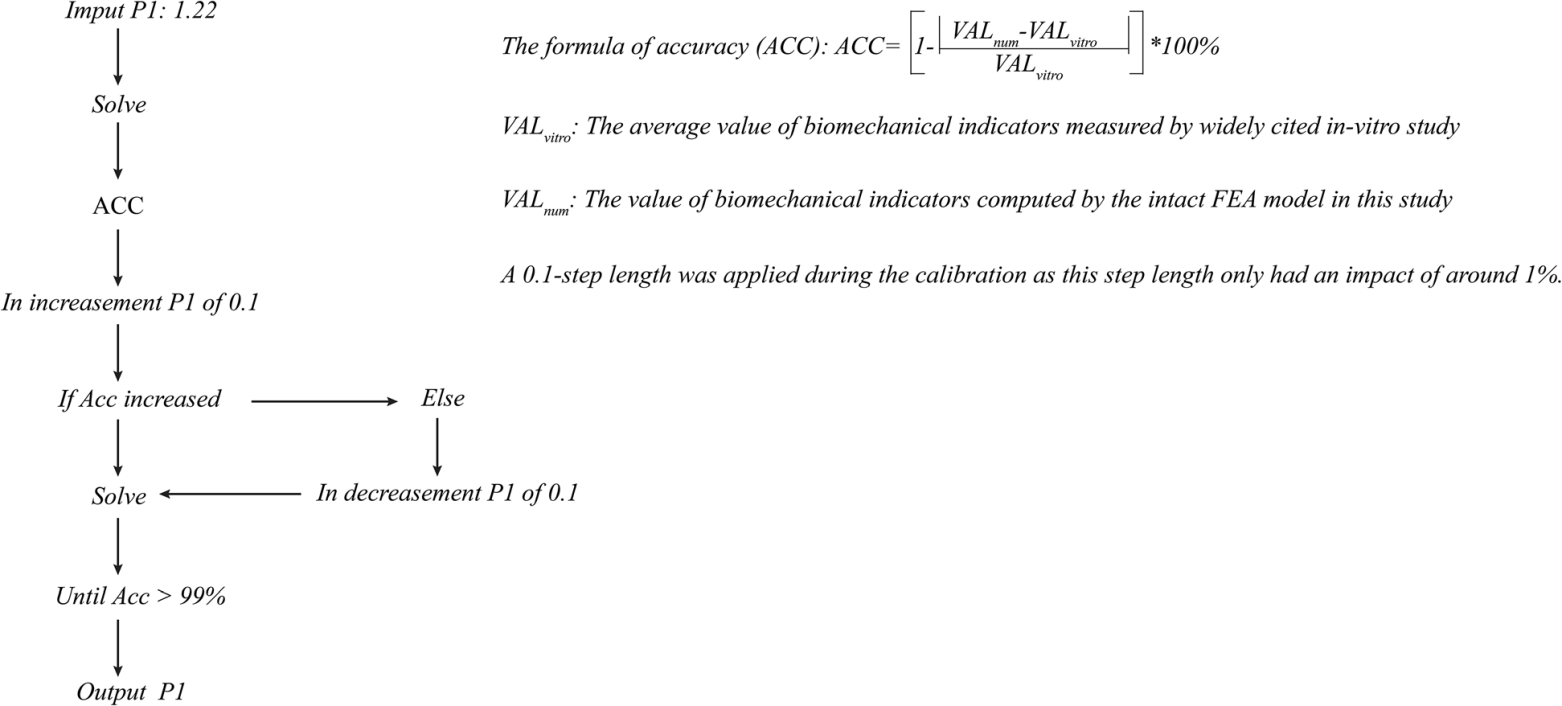
Calibration algorithm for the relative nucleus position

The calibration process was accomplished by adjusting the value of P1, estimating the RoM under a moment of 10 Nm and flexion–extension conditions, and finally comparing our FEA model results to the results of a widely cited in vitro study [39, 40]. The nucleus, ligaments and facet joints were suppressed during this process. The calibration algorithm was presented in the Fig. 5.

#### Model validation

The model was validated by evaluating the difference between different biomechanical indicators, including the RoM, the intradiscal pressure (IDP), the facet contact force (FCF), and the disc compression (DC) from the current FEA model (with nucleus, ligaments, and facet joints) and those from the in vitro studies to determine if the two measured and calibrated factors reflected real biomechanical indicators under different loading conditions and to determine whether the calibrated FEA model can be used in subsequent studies [41–43]. Specifically, different loading conditions were used for the computation of different model validation indicators. The IDP and FCF were computed under 7.5 Nm moments, the RoM was computed under a 10 Nm moment with a 100 N compressive force, and the DC was computed under 1200 N compressive force.

#### Evaluation of computational efficiency

The computational time of different FEA models were recorded to investigate if the smoothened model constructed with the fitted curve could increase the computational efficiency compared with that of the reconstructed model. During this process, FEA models were modified on the basis of the measured and calibrated nucleus relative position and cross-sectional area ratio, the same boundary and loading conditions were set in these two models, and all the analyses were accomplished in the same workstation.

## Results

### Descriptive data of the MRI measurement

MRI data from 43 subjects (average age of 25.6 ± 4.3 years) were included in the current study. To avoid the influence of gender differences, only male MRI data were collected for the following FEA model based on the imaging data from a 24-year-old male volunteer. The P1 and P2 values then calculated from the measured values and used in the FEA model (P1 = 1.22; P2 = 38%).

### Homogeneity test

The kappa values for the interobserver disc degenerative classification were in the 0.67–0.77 range, and the Cronbach’s α values for D1, D2, A1, and A2, all of which were measured by different observers, were greater than 0.95. These values indicated acceptable interobserver homogeneity (Table 1 and Table 2) [35, 36, 44].

**Table 1:** Homogeneity test of measured values

	Average values	Cronbach’s α
D1	8.87 ± 1.51	0.97
D2	7.29 ± 1.37	0.96
A1	1761.0 ± 206.1	0.96
A2	671.4 ± 123.8	0.98

**Table 2:** Homogeneity test of observers

Observers	Kappa values
1&2	0.72
2&3	0.77
1&3	0.67

1, 2, and 3 stand for three observers in this study, 1 and 2 are senior spine surgeons and 3 is the musculoskeletal radiologist

### Calibration of the relative nucleus position

**Fig. 6:**
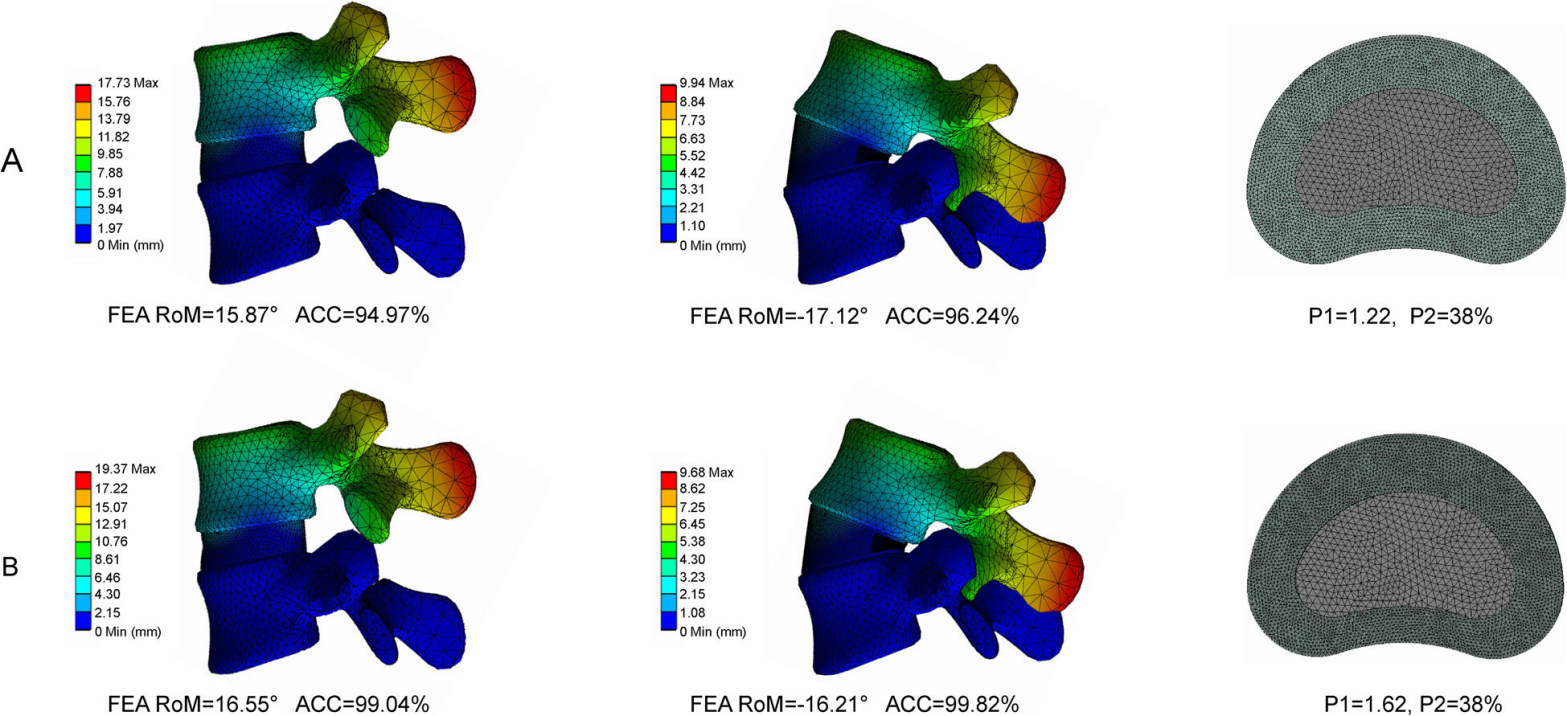
Variations in the RoMs before and after model calibration. **A** RoMs before the calibration of the relative nucleus position. **B** RoMs after calibration of the relative nucleus position

The definition of accuracy is shown in the Fig. 5, with the sensitivity of the data adjustment tested prior to calibration. We found that the accuracy under flexion–extension reached 99% when the P1 value was calibrated to 1.62. This value was confirmed and used during the model validation process (Table 3, Fig. 6).

**Table 3:** Calibration of nucleus relative position

Cadavers study (10 Nm)	P1	Calibration data (°)	Computational accuracy (%)
Flexion (16.71°)	1.12	15.49	92.69
	1.22	15.87	94.97
	1.32	16.13	96.53
	1.42	16.31	97.61
	1.52	16.42	98.26
	1.62	16.55	99.04
Extension (− 16.24°)	1.12	− 17.12	94.58
	1.22	− 16.85	96.24
	1.32	− 16.51	98.34
	1.42	− 16.39	99.08
	1.52	− 16.30	99.63
	1.62	− 16.21	99.82

### Model validation

**Fig. 7:**
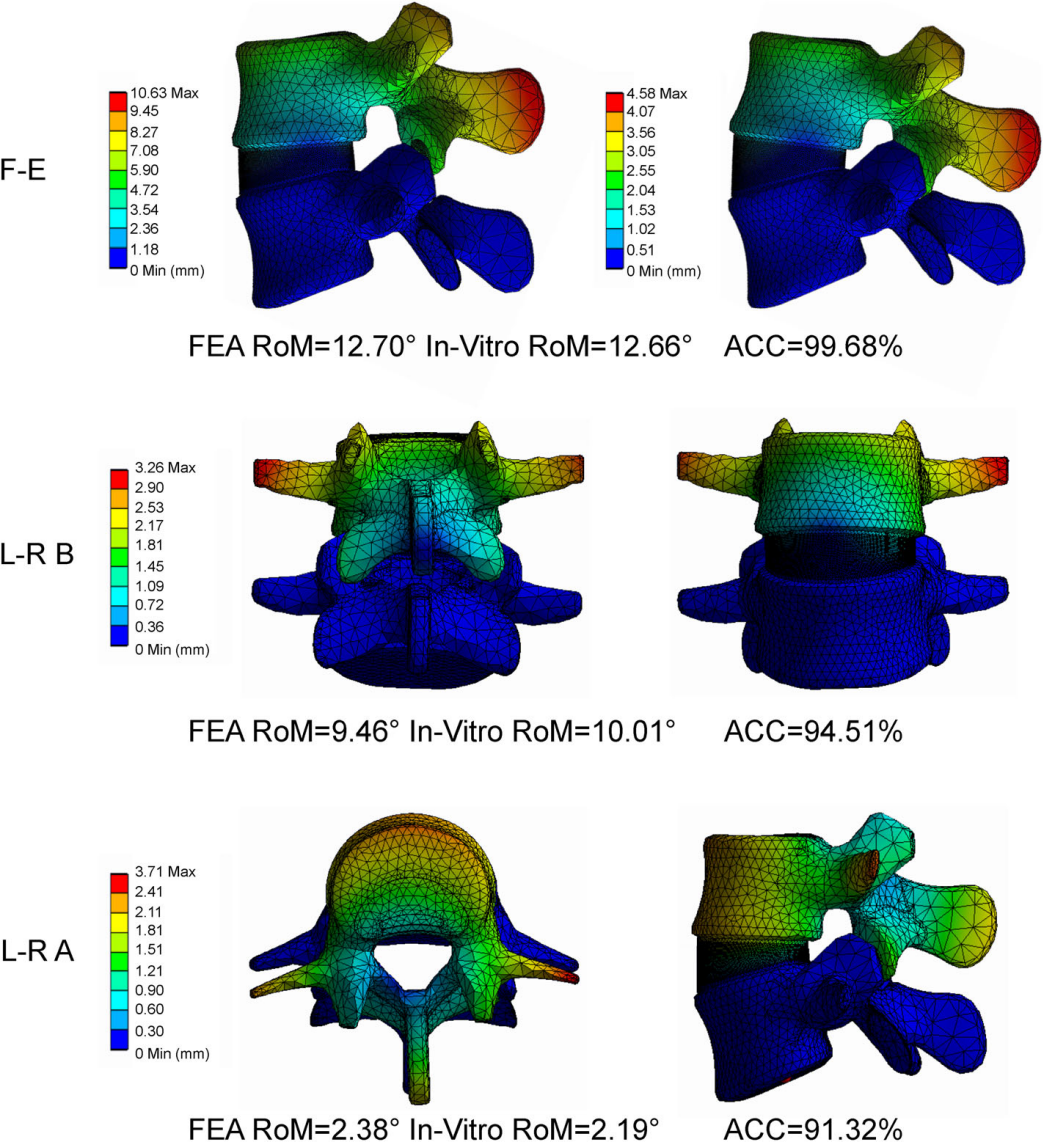
Validation of the calibrated model by comparing the RoM results F-E=Flexion-extension; L-R B=Left-right bending; L-R A=Left-right axial rotation

**Fig. 8:**
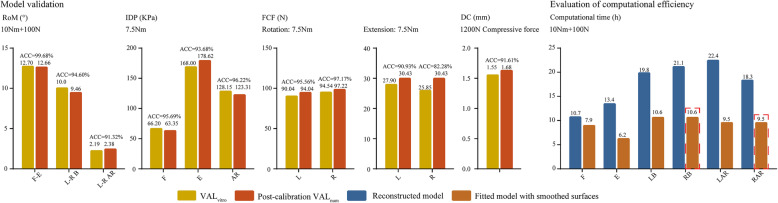
Model validation and computational efficiency evaluation. *F* flexion, *E* extension, *B* bending, *AR* axial rotation, *L* left, *R* right, *ACC* computational accuracy

The validated model was modified using the calibrated relative nucleus position. We found that the accuracy of RoM was higher than 90% under all loading conditions, including flexion-extension, left-right lateral bending, and left-right axial rotation, and was higher under the flexion–extension condition (Fig. 7) [41]. The accuracy of the IDP the FCF and the DC were also higher than 90% under almost the loading conditions except for the right side of FCF under extension condition (Fig. 8) [41, 42]. In which, the value of computational accuracy was 82.28% and the difference between our computational result and the average value from in-vitro study was still obviously less than one standard deviation [43].

### Variation of computational time

The computational time of the model with the fitted curves dramatically reduced compared with that of the reconstructed one. Additionally, in the fitted models that were strictly symmetric along the sagittal plane, the computation under lateral bending and axial rotation conditions was accomplished only in one rather than in two directions. Thus, the computational time could be reduced further, and the total time spent in the fitted model decreased by 67.6% compared with that of the reconstructed one.

## Discussion

In this study, the computational accuracy and efficiency of the FEA model constructed via an innovative method (which could reduce the model’s irregular surfaces and structures (i.e., different from the ‘model reconstructive method’) and preserve the original structure outline (i.e., different from the ‘standard geometry method’)) with measured and calibrated nucleus relative position and its cross-sectional area ratio were explored to optimize the biomechanical research on spinal FEA.

A contradiction was observed during the construction of the FEA models; specifically, macroscopic irregular structures in the models constructed with the ‘reconstructive method’ inevitably reduce the repeatability of FEA, and irregular surfaces in these models increase the incidence of mesh deviation, the related computational burden and the risk of computational divergence [5, 11, 15, 16]. Oversimplified FEA models constructed with the standard geometry method eliminate almost all the structural details and possibly reduce the credibility of FEA [4, 17–19]. By contrast, the models constructed with the presented method (i.e., using the fitted curves to depict the external contour of model structures) could result in the balance between the repeatability and structure details of FEA models. Specifically, the computational burden decreased dramatically not only because the incidence of mesh distortion decreased in the replacement of irregular surfaces and structures but also because one side loading condition could be omitted in the simulation of lateral bending and axial rotation in the current FEA model, which was strictly symmetric along the sagittal plane (Figs. 1 and 4). Additionally, in terms of the reliability of FEA, aberrant stress distribution could be often observed in irregular structures, and the replacement of these structures by the smoothened one could optimize the credibility of FEA. Given that most of the structural details of the current FEA model were preserved, its computational results should be more reliable than that of the models from the standard geometry method.

We explored several methods that defined the relative nucleus position and its cross-sectional area ratio, but there were still several issues with regard to our FEA models [23, 29, 39]. The data from previous studies could not be directly used in our model construction owing to individual differences and the lack of consistent standards [23, 29]. For example, the previously published nucleus position, wherein its center was located 3.5 mm toward the posterior of the disc, yielded a D2 value of less than 2 mm, which is obviously less than the minimum value measured in our study. Furthermore, the application of this value to the models constructed using imaging data from short volunteers may result in an impossible situation wherein part of the nucleus lies outside the disc’s boundary. The lack of consistent definition methods also leads to repeated attempts at model construction and validation. Finally, we can only define the relative nucleus position as being ‘slightly posterior’ to the center of the disc, such that the lowest accuracy of our model was lower than 70%, which needs to be improved further, even though the model validation process was verified in our previously published studies [37, 38]**.** Therefore, the calibration of the relative nucleus position and the investigation of a reliable ratio to define the above indicators are vital for improving the accuracy of FEA, a widely used research method in spinal biomechanical studies [8, 11, 45].

The reduced T2 signal in the MRI data is closely related to disc degeneration and is generally selected to measure such a pathological change [30, 33]. The homogeneity test is important for ensuring the accuracy and credibility of the study, as it is based on subjective observer measurements [35, 44]. While the kappa values between observers 1 and 3 are excellent (> 0.75), the rest are only acceptable (> 0.6). Such a phenomenon may be attributed to the small sample size and strict inclusion criteria of this study. These two constraints highlight that the slight interobserver differences can lead to obvious variations in the kappa values. Furthermore, although there is a certain degree of difference between the measured and calibrated relative nucleus positions, the modeled RoMs that are constructed from the measured nucleus position are still quite similar to the values from the in vitro study, with excellent accuracy values attained (94.97% under flexion and 96.24% under extension, Fig. 6). The model constructed from the measured values also simulates real biomechanical indicators, such that the model calibration process further improves the computational accuracy based on the MRI measurements.

Besides, the reasons why we chose P1 rather than P2 as the calibrated factor should also be clarified. In Fig. 6, when P1 = 1.22 and P2 = 38%, the overall stiffness of the disc was relatively larger than the mean value from an in vitro study in flexion and smaller than which under the extension condition. Therefore, the objective of the model calibration was to increase the range of motions (RoMs) in flexion and decrease RoMs under the extension condition. In the manuscript, the increase in the cross-sectional area ratio of the nucleus causes a decrease in the overall stiffness of the intervertebral disc (IVD). As a result, RoMs under each loading condition increased simultaneously, and this change was obviously contradictory to our model calibration objective. By contrast, the stiffness of the IVD could be adjusted in different directions (i.e., enhance the stiffness of the anterior part of the IVD and decrease which of posterior IVD at the same time) by changing the relative position of the nucleus. P1 (the relative position of the nucleus) rather than P2 (the cross-sectional areas of the nucleus) was selected as the model calibration factor.

Notably, the nucleus itself was suppressed during the model calibration process in the current study, even though the measured and calibrated values were closely associated with the nucleus. This is because R1 and R2 have an important impact on both M1 and M2 and their resultant RoMs. The variation in the RoMs is more likely due to the change in the approximately quadrangular annulus areas caused by the change of relative nucleus position and its cross-sectional area rather than the nucleus itself. The nucleus, ligaments, and facet joints were therefore suppressed during the model calibration to investigate this factor individually. The FEA study results indicated that the retrodisplacement of the nucleus improved the accuracy during the calibration process. Furthermore, the posterior structures overlapped in the model calibration under the extension condition. This phenomenon is not indicated by the computation error; rather, it is caused by the omitted contact types between the bone structures. Therefore, spatial positions are independently calculated when facet cartilages have been suppressed (Fig. 6).

In this study, the nucleus positions before and after calibration clearly changed (Fig. 6). We hypothesized that this change may originate from changes in body positions. The MRI measurement was completed in the supine position, whereas the loading conditions in the biomechanical studies were based on the standing position. Considering that the position of a nucleus cannot be changed once it was determined in the model construction process and that MRI data can be obtained in only the supine position, we believe that this model calibration is an effective method to calibrate this difference caused by body position and improve the computational accuracy.

Differences still exist between the RoM in the current FEA study and the widely cited in vitro study; these differences may be due to the suppressed structures on the RoM, even though the measurement and calibration of the relative nucleus position and its cross-sectional area ratio increase the accuracy. This defect may also affect the accuracy because the ligament definition also lacks a standard method. There are no published in vitro RoM values that have been computed from models with the ligaments, facet joints and nucleus removed and with intact bone structures under lateral bending and axial rotation conditions. Hence, we are unable to calibrate the FEA model under these loading conditions. This defect can provide a good explanation for the lower accuracy under lateral bending and axial rotation conditions.

Due to the issue that RoM predictions alone are insufficient to validate models for predicting mechanical contact parameters, multi-indicator model validation was necessary to evaluate the credibility of the measured and calibrated factors [45]. In addition, although 10 Nm moments were widely used in published finite element studies [5, 10, 13, 46], model validations under smaller moments were also necessary for the wider application of these measured and calibrated factors [12, 47, 48]. Hence, IDP and FCF under 7.5 Nm moments and DC under 1200N compressive force were computed as validation indicators [41, 42].

In this process, it is also worth noting that we did not determine the value of FCF from an in vitro study of the L4–L5 segment; hence, FCF from a widely cited in vitro study of the L3–L4 segment was selected as the standard value for the validation of FCF [43]. Computational FCF results indicated that the L4–L5 segment was slightly larger than the in vitro values (Fig. 8), and this result was consistent with the report that the facet contact force gradually increased from the cranial to the caudal side [12]. Despite these defects, we still believe that the difference in the model validation is acceptable because the lowest accuracy value computed in the current study (among the RoM, IDP, and FCF) is greater than 90% under almost all of loading conditions. Therefore, the measured P2 and calibrated P1 values in our subsequent FEA studies can be used to increase computational accuracy.

The current study still faces some limitations. The ratio measurement of nucleus position is based on cross-sectional areas in a specific two-dimensional plane rather than the three-dimensional volume, such that the models do not capture differences in the lumbar lordotic angle. For example, obvious changes in the disc volume can be observed in the models with the same cross-sectional area ratio and different lordotic angles. Furthermore, the definition of the ligaments was accomplished based on our observer measurements and did not conform to a uniform standard, even though ligaments play a significant role in the maintenance of lumbar stability and are a key index in RoM [49]. Therefore, the definition of ligaments should be investigated and calibrated in future studies to further develop more accurate FEA models.

## Conclusion

The measured and calibrated relative nucleus position (P1 = 1.62) and its cross-sectional area ratio (P2 = 38%) in lumbar models constructed with smoothened surfaces could increase the accuracy and efficiency of FEA, and be beneficial for further spinal biomechanical studies.

## Data Availability

All the data of the manuscript are presented in the paper.

## Abbreviations

DC: Disc compression
FCF: Facet contact force
FEA: Finite element analysis
IDD: Intradiscal pressure
RoM: Range of motions
